# Virtual reality assessment of walking in a modifiable urban environment: a feasibility and acceptability study

**DOI:** 10.1038/s41598-023-32139-w

**Published:** 2023-04-11

**Authors:** Katrina Oselinsky, Amanda N. Spitzer, Yiqing Yu, Francisco R. Ortega, Laura H. Malinin, Kelly A. Curl, Heather Leach, Dan J. Graham

**Affiliations:** 1 Department of Psychology, College of Natural Sciences, Colorado State University, Fort Collins, CO USA; 2 Department of Community and Behavioral Health, Colorado School of Public Health, Fort Collins, CO USA; 3 Department of Computer Science, Colorado State University, Fort Collins, CO USA; 4 Department of Design and Merchandising, College of Health and Human Sciences, Colorado State University, Fort Collins, CO USA; 5 Department of Horticulture and Landscape Architecture, College of Agricultural Sciences, Colorado State University, Fort Collins, CO USA; 6 Department of Health and Exercise Science, College of Health and Human Sciences, Fort Collins, CO USA

**Keywords:** Psychology and behaviour, Disease prevention, Lifestyle modification

## Abstract

Physical activity is known to be one of the most health-beneficial behaviors, and salutogenic design modifications to the built environment can facilitate increased physical activity. Unfortunately, it is not often clear in advance which environmental and urban design implementations will generate increases in activities such as walking, and which will have little impact or even reduce walking. The present study tested the feasibility and acceptability of a virtual reality (VR) model for pre-testing urban designs for their impact on walking. Using a wearable VR head-mounted display/computer, young adults (n = 40) walked freely through a large indoor gymnasium, simultaneously walking through a virtual model of an urban streetscape that was designed to be modifiable and allow for testing impacts on walking of various changes to the urban environment. The majority of participants found the experience to be acceptable: pleasant and nonaversive, and they walked freely through the VR model for approximately 20 min, on average. Using modifiable VR models to pre-test built-environment changes for their impacts on walking behavior appears to be a feasible and acceptable approach and worthy of continued research investigation.

## Introduction

Physical inactivity is one of the most important contributors to the global burden of noncommunicable disease, accounting for over five million premature deaths each year^1^. Population-level increases in physical activity (PA) can greatly reduce the impact of cancer and other chronic diseases^1–4^. Specific estimates attribute 10% of the disease burden associated with both breast and colon cancers to physical inactivity as well as a similar proportion of all premature mortality and well over $50 billion in annual global health care costs^1,5–7^. Unfortunately, most U.S. adults are highly sedentary^8–10^; only 27.1% report daily PA^1^. Walking is one of the most accessible, affordable, and achievable forms of PA. It is safe, presenting a very low risk of injury for nearly all individuals^11^, and requires no specific skills, training, or equipment. Thus, interventions promoting walking have the capacity to impact a large number of people in many locations, with a significant potential public health benefit.

Specifically, salutogenic design interventions in the built environment have the capability to promote walking on a large-scale. Salutogenesis is a human-health concept that orients resources toward promoting and maintaining physical and mental health as an alternative to focusing on disease treatment^12^. Salutogenic design is an evidence-based approach to designing features and qualities of the built environment to promote user health and wellbeing^13^. For example, places that lack pedestrian-friendly infrastructure (e.g., sidewalks, crosswalks) inhibit safe and comfortable walking and adding this infrastructure can increase walking, thereby encouraging users to engage in healthy behaviors^14^. Health behaviors including walking are influenced by factors at every level within social ecological models^15^, but the environmental design level has significant potential to benefit public health due to the ability of environmental changes to impact all individuals who interact with them. Thus, intervening on the environment via sustainable urban design is a high-leverage strategy for promoting PA and subsequent health outcomes. Although alterations to the built environment may be a promising avenue by which to increase PA, Bozovic and colleagues^16^ found disagreement among professionals in fields related to urban planning and public health concerning how users experience aspects of environments that either help or hinder walking, suggesting more research is needed.

Currently three methods (correlational studies, quasi-experiments, and photo-elicitation studies) are most commonly used to identify relationships between the environment and PA.

### Method 1: Cross-sectional studies

Cross sectional studies primarily measure associations between PA and various geographic locations or features^17–39^. This research can identify associations between environmental design features and PA (e.g., more walking occurs in a neighborhood that contains a walking path, therefore, the presence of a path *is associated with* more walking), but such research cannot prove causation. Cross-sectional study designs are limited; they cannot tell planners whether making a specific addition or modification will increase walking.

### Method 2: Quasi-experiments

Quasi-experiments assess PA before and after some event. One type evaluates PA before an environment undergoes specific design alterations and then measures PA again after that alteration (e.g., when a new trail is added to a neighborhood)^14,40,41^. These study designs are resource-intensive and not always feasible. Full-scale implementation of environmental modifications is costly and may require more time and money than researchers and local governments are able to invest. Therefore, pursuing one environment change will often preclude the pursuit of another; consequently, meaningful comparisons between physical environmental interventions cannot be completed. Even if there are enough resources to complete these changes, randomly assigning individuals to experience various environmental changes is often impossible.

Another type of common quasi-experimental design in built environmental research into PA measures PA before and after an individual relocates to a new neighborhood^42–44^. These studies have important limitations as well, particularly regarding internal validity: it is difficult to determine which specific environmental difference(s) between the old and new neighborhoods led to any observed changes in PA as neighborhoods tend to differ on many dimensions.

### Method 3: Photo elicitation studies

A third common study design to examine the environment/PA relationship is using photos. The photo-voice method uses interviews to discuss photos that participants captured of places they do/do not like to walk^45–47^. Less commonly, experimental design methods are used with modified photos, videos, and 2D simulations of environments (e.g., adding/subtracting elements such as greenery)^48,49^. Participants report their preferences, perceptions, and/or activity intentions in these modified environments. Researchers use this data to infer environmental attractiveness, the degree to which individuals favor the environment^48^. These experimental studies can address causation, but the outcomes are not optimal. Intentions do not always correspond to PA *behavior*^50^; thus, researchers must measure PA behaviors and decisions themselves to identify the most useful environmental changes.

To summarize, three primary barriers to progress in this field can be identified. First, neither cross-sectional designs nor quasi-experiments provide strong support for causation. Second, quasi-experiments are resource-prohibitive. Third, the intention/behavior gap demonstrates that conclusions from respondent reports based on 2D representations of environments do not necessarily translate into knowledge about walking decisions^50^.

To advance the interdisciplinary field of salutogenic design, we must test methods of experimentally evaluating the impacts of environment changes on PA behavior, without the extensive monetary and time investment required to alter the built environment. Such causal evidence will advance understanding of how to increase the potential for salutogenic design interventions in the built environment to promote PA. Immersive virtual reality (VR) technology has the capability to address these barriers and advance the field. The novel use of immersive VR technology in this context allows us to add and subtract environmental features in a controlled way, enabling random assignment of participants to environments that differ only on a single dimension of interest. VR technology can allow us to make causal statements about the impact of salutogenic environmental design changes on PA.

VR technology is designed to create immersion, meaning the illusion of presence in an artificial environment^51^. Presence is defined as the perception of “being there,” as if the user is in a physical environment^52^ while immersion involves several features, including: (1) presence, (2) the illusion of self-embodiment, perceiving their own body in the virtual space, (3) the perception of physical interaction with the virtual space^51^. A related construct is visual realism, which represents the degree to which the visual experience of the virtual environment mirrors real life visual experiences^53^. Visual realism concerns the form and lighting of virtual elements, and high visual realism increases presence^54^. Due to the environment in which humans have evolved, our brains similarly process real stimuli and mediated representations such as the virtual stimuli included in the present study’s VR models^55^. Thus, individuals are able to become highly immersed in VR environments, and behavior can represent what individuals do in real life. There are many examples of how behavior in VR translates to the real world, including the effectiveness of VR-based exposure therapy^56^. In fact, many training programs use VR to help people prepare for high-stakes real-world situations, such as performing surgery and donning aircraft life preservers^57–63^. Given the lower costs (compared to real-world construction costs) and high ecological validity of VR approaches, researchers have begun to explore its potential to better predict user outcomes in environmental designs, particularly with respect to wellbeing^64–67^.

Most crucially for the study of salutogenic environmental modification and walking behavior, there is evidence that pedestrian behavior in VR looks like walking in real life with respect to evaluation of safety^68^ and road crossing behavior^68,69^. Additionally, aspects of perception related to walking have been shown to be comparable in VR as to real life such as judgments of distance^68,70–72^. Blind navigation of a previously perceived route is worse in VR^71^, but this task is highly manufactured and lacks ecological validity. Lastly, subjective impressions of VR environments have been demonstrated to be equivalent to those of their real-world counterparts^73,74^, which is fundamental to researching environmental modifications in VR.

There is much literature regarding the physical mechanics of walking in VR. One study found differences in walking speed, walking mechanics, and heart rate between a task performed in VR and the same task performed in real life^75^; however, in this study, participants in VR utilized a unidirectional treadmill, turned using a joystick, and the VR display was monitor-based (not an immersive head-mounted display). Another study reports that preferred walking speed in VR is reduced compared to that in real life, but preferred walking speed was assessed on a treadmill, which may cause VR users to proceed more cautiously since they are unable to see where they are in relation to the treadmill^76^. Also, while using VR, participants in this study walked through a highly vegetated park-like VR environment, and vegetation has been shown to decrease walking speed^77^. Lastly, walking in VR along what appeared to be an elevated beam resulted in the expected behavioral (decreased speed) and physiological reactions (e.g., increased heart rate) as compared with walking along a beam at ground level^78^. Recently, researchers have begun using VR to investigate environmental influences on walking behavior. Some recent studies have used VR methodology as an immersive version of photo-elicitation studies. In these, participants are asked to evaluate walkability of VR environments that move automatically, (i.e., participants do not walk through these environments themselves)^79,80^. One unpublished dissertation study used an immersive room (CAVE) that automatically moved to simulate walking through different urban configurations to investigate their impact on pedestrian perceptions^81^. Participants were allowed to stand still or walk around the room while the simulation moved around them, irrespective of their own movements. Additionally, VR has been used to measure perceived safety of various environments through self-report^82–84^ and physiological responses^83^ using motionless VR environments. While impressions formed in VR environments are more similar to those formed in their corresponding real-world environment than are impressions formed using photographs^84^, these studies still do not investigate walking behavior as participants do not meaningfully walk to navigate the VR environments. One pilot study explored an omnidirectional treadmill as a possible methodology for participants to walk in VR^85^. This treadmill requires users to slide against its surface. The authors note issues with this walking method, including the “unnatural posture” participants had to employ^85^. Another pilot study compared two omnidirectional treadmills (including the one from the previous study) with walking in place to locomote in VR^86^. No method was identified as preferable though they differed on various dimensions, including perceived safety and required energy.

Overground walking may provide participants the ability to meaningfully and comfortable walk in virtual environments. There is a limited volume of recently published studies describing the relationship between walking kinematics in the real-world and overground walking, and we have seen mixed results here. Individuals adjust their walking mechanics to respond to VR environmental demands while on a treadmill^87^ but adjust more so more during overground walking^88^. Some studies showed that there is no significant difference between walking in a virtual environment and in a real-world situation, and people are able to adapt to the virtual environment^72,89^. Moreover, Canessa and colleagues^72^ found stride length was comparable in VR and real life although step cadence, count, and speed differed; however, the walking task examined in their study required participants to walk a straight short distance before making a half turn and walking back, so results may not be applicable to walking behavior that is more prolonged and/or naturalistic. A final study found that there is a significant difference between walking velocity in the virtual environment and in the real-world, and this difference was not reduced over time^90^. Moreover, there is some nuance to overground walking behavior when the conditions of the virtual environment or physical environment do not align. Individuals follow virtual environment restrictions when they correctly identify them as such, but at times, they also deviate due to curiosity about the ability to walk through purely virtual obstacles^91^. In other words, under certain conditions people react to virtual conditions as they do in real life. When participants are given visual directional prompts in a virtual environment which does not match real-world constraints, they closely follow those prompts with small steps, indicating that participants do not feel safe in the environment^92^. Still, this research cannot tell us about everyday walking decisions at the urban scale, such as the choice to walk to a location instead of driving or to walk further than required. In order to measure the causal impact of environmental modifications on actual adoption of walking behavior, we have developed an innovative methodology. The purpose of this methodology is to immerse participants into a city-scale modifiable virtual environment and allow free movement in the environment via overground walking. While VR technology has been used in walking research^72,75,76,79–92^, we propose a novel use affording participants the ability to physically walk around a large urban environment without the use of a laborious omnidirectional treadmill. Our study set-up allows researchers to address novel questions such as how environmental modifications affect decisions regarding whether to walk and for what duration. These questions are central to walkability research, which aims to increase the number and duration of walking trips in a community through the built environment. Therefore, for the present study, features of walking are less focal than other features such as immersion, comfort, and useability.

**Figure 1:**
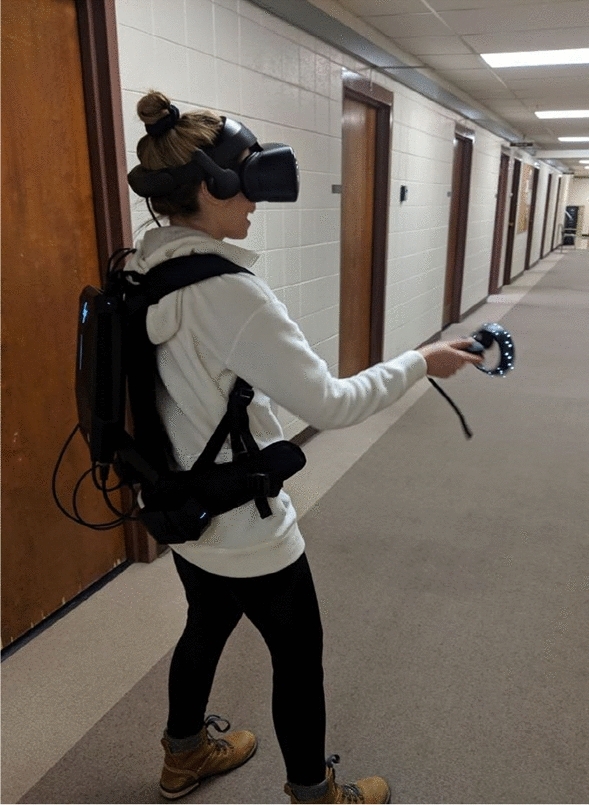
One of the authors wearing VR HMD and backpack computer.

To achieve this goal, we use virtual reality (VR) head mounted displays (HMDs) and backpack computers (Fig. 1). As noted, overground walking induces larger mechanical walking adjustments when required by the environment presented in VR than treadmills, meaning individuals kinematically respond more to environmental cues^88^. Due to these differences between treadmill walking and overground walking, for testing the effects of environmental changes on overground walking, it would be preferable to test these effects on overground walking, rather than walking on treadmills. While there are wireless HMDs (e.g., Oculus Quest), we used a wired VR HMD because it provided additional features, such as eye-tracking capability (not used for the present study) and higher resolutions. Using backpack computers, VR HMDs enable participants to walk freely while providing real-time objective PA data. Thus, we do not rely on intention or self-reported PA but are able to test the causal effects of environment change on measured PA itself.

Still, HMDs have their challenges. While HMDs were found to be more usable during a walking task and to elicit more presence in the simulation than a monitor-based option when completing identical tasks, they were found to place a larger mental demand and workload on their users and induced more frustration^93^. Researchers using HMDs should evaluate the perceived task difficulty to ensure that they are not placing too high of a workload on participants. Additionally, early in HMD development, users felt the equipment was uncomfortable^94^. Though contemporary HMD devices are more comfortable, it is still important to assess equipment comfort in any HMD setup. Evaluation of controller usability is also useful as the method of VR controller training has been identified as a meaningful decision in environmental research using VR^94^. Relatedly, Birenboim and colleagues^85^ note that participants who are new to VR may attend to the technology instead of the study task; essentially, they have not yet adjusted to VR. Lastly, Kourtesis et al.^95^ recommend a maximum length of 55–70 min for VR HMD in order to avoid cybersickness. Cybersickness is used to describe the adverse symptoms of VR, including nausea, dizziness, and eyestrain^96^. Presence of symptoms can depend on software qualities and chosen hardware^95,97^. To better understand whether our proposed strategy for assessing the effects of environmental changes on walking behavior using VR HMDs and backpack computers was feasible and acceptable to participants, it was necessary to pilot test our methodology and simulation.

**Figure 2:**
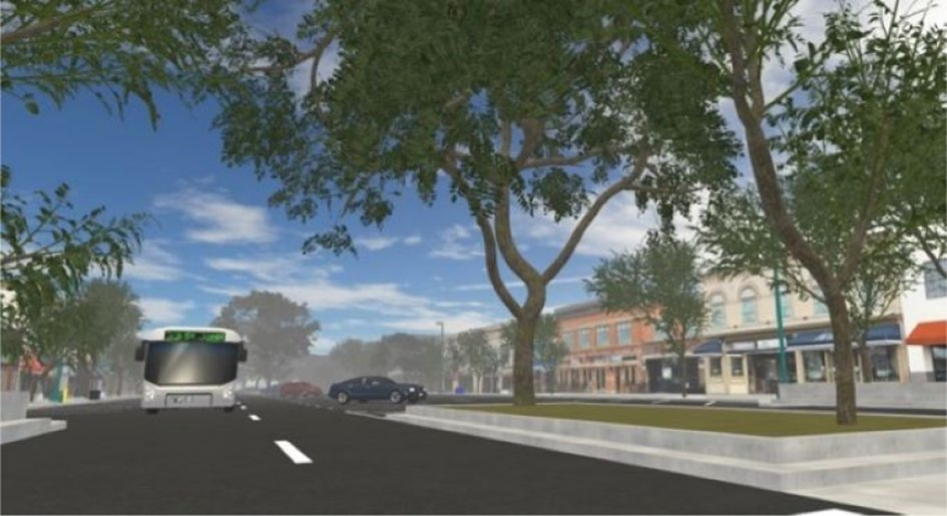
Example scene from modifiable VR environment used in pilot research.

**Figure 3:**
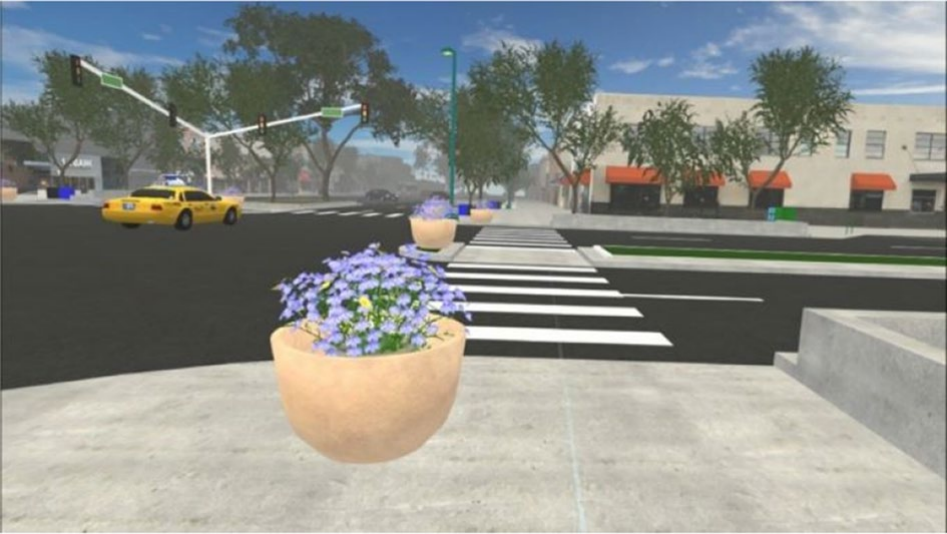
Example scene from modifiable VR environment used in pilot research.

**Figure 4:**
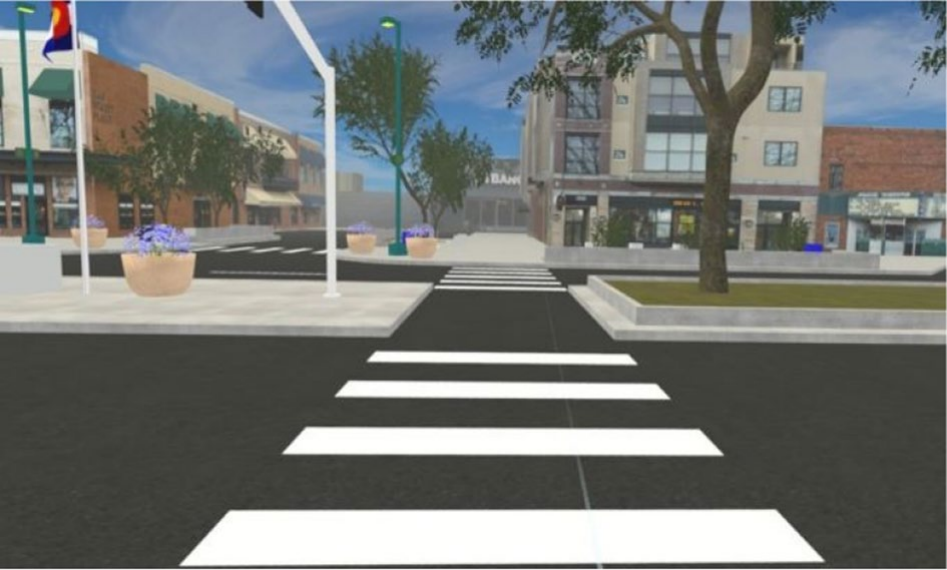
Example scene from modifiable VR environment used in pilot research.

The present study serves as a test of the feasibility and acceptability of our novel methodology. Participants walked through a modifiable VR model of four city blocks in the historic downtown area of a mid-sized city in the western US (Figs. 2, 3, 4), while wearing a backpack computer and VR HMD and walking at their own chosen speed through a large open indoor gymnasium. The aim is to investigate the feasibility of using this methodology to enable participants to walk freely as opposed to using a treadmill or other restrictive technologies, as well as the acceptability of this approach from the perspective of the research participant (i.e., is the experience of walking through the designed environment realistic, comfortable, engaging, etc.). If feasible and acceptable, this methodology could afford researchers a novel avenue for experimentally assessing the impact of environmental modifications on PA.

## Methods

### Participants

**Figure 5:**
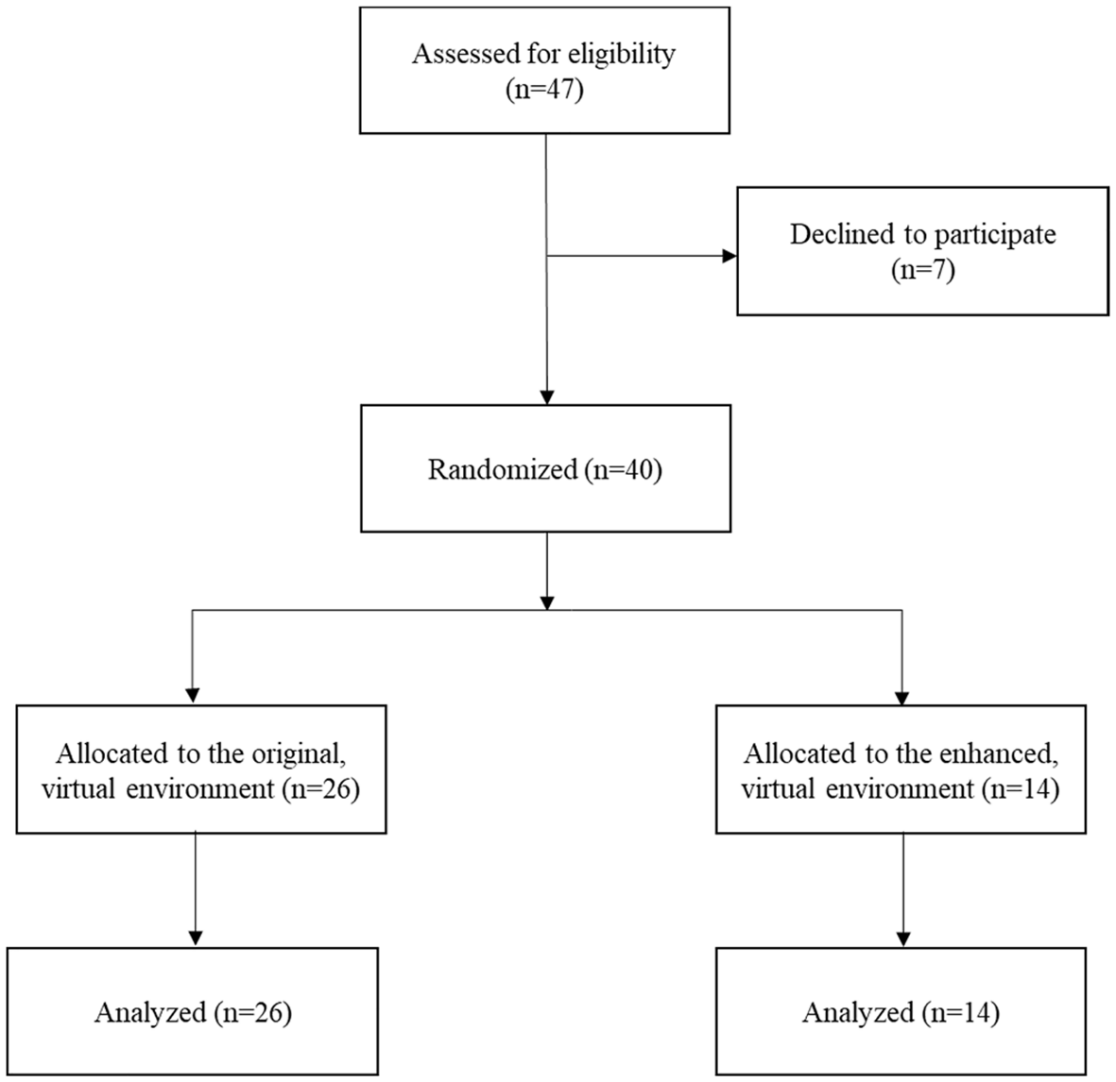
Study phases flow diagram.

Convenience sampling from the spring 2019 research participant pool at a large university located in the western United States produced 40 undergraduate study volunteers (Fig. 5). To be eligible to participate, students needed accurate or corrected vision via soft contact lenses or eyeglasses. Participants’ average age was 18.7 years (SD = 1.38), and 60% of the participants were female. Participants were compensated for their participation via course credit. Prior to data collection, the research team obtained approval from the host university’s Institutional Review Board, and all participants were required to sign an informed consent document before engaging in study activities. This study was conducted in agreement with the Declaration of Helsinki and later amendments.

### Apparatus/measures

#### Virtual reality simulation

Our virtual environment is a recreation of a portion of an existing historic downtown area of a mid-sized US city. To build this virtual environment, the research team utilized the 3D modeling program SketchUp (2015, Version 16.0.1) to convert digital assets of a base model. The Unity Game Engine (2017, Version 2017.3.0) and Blender (2017, Version 2.79) were used to virtually recreate the urban environment for VR. All SketchUp models underwent mesh decimation to reduce the total geometric complexity for the scene, and key meshes such as sidewalks, brick, asphalt, dirt, and grass were re-textured for added realism. Trees and vegetation, sound effects, atmosphere, lighting, and moving cars were added. Additional graphics optimizations such as adaptive level-of-detail and occlusion culling allowed for navigation of the scene while meeting the performance demands of real-time VR rendering. In the future, this simulation may be used in an experimental study, manipulating street-scale factors, such as the amount of greenery, to determine their effects on walking behavior.

#### VR apparatus

Data collection occurred indoors in an empty gym that was approximately 275 × 148 feet (83.82 × 45.11 m) to ensure adequate space for participants to explore the simulation. The HP Z VR Backpack G1 Workstation computer with Intel^®^ Core™ i7-7820HQ running Windows 10 Version 1809 was paired with the Samsung HMD Odyssey Windows Mixed Reality Headset (Model XE800ZAA-HC1US) with Motion Controllers (Models AA-HCIHULB & AA-HCIHURB). Using a backpack computer provided participants the ability to walk freely overground; as discussed, overground walking evokes greater mechanical responses to environments^88^. We chose the Samsung HMD Odyssey because its display is of a high resolution, and it is capable of eye-tracking data (not used for the present study). All adjustable parts of the backpack and HMD were fitted for participant comfort (i.e., backpack shoulder straps, backpack waist strap, HMD occipital cradle, and the distance between the two eye pieces on the HMD). Participants were provided one hand controller which enabled them to rotate the environment if necessary (i.e., if they wanted to keep moving in the same direction in the simulation but had to walk in a different direction in the physical environment, as when approaching a wall—participants could physically change their direction within the gym, while continuing to walk in the same direction in the VR environment).

#### Post-experience survey

After completing the VR portion of the study participants completed a 27-item survey probing the following constructs: VR adjustment (1-item), cybersickness (4), visual realism (1), equipment comfort (1), controller usability (1), immersion (1), perceived safety (2), environment attractiveness (1), and perceived task difficulty (1). VR adjustment was operationalized by having participants identify the duration of time it took to adjust to the VR environment on an anchored 5-point scale. Questions concerning cybersickness asked participants to rate on a 1–5 scale in which 1 = none at all and 5 = a great deal, the level to which they experienced the following sensations while in the VR simulation: discomfort, nausea, dizziness, and eyestrain, which are all symptoms of cybersickness^96^. The remaining constructs were operationalized by asking participants to rate their experience while in the virtual environment on a 5-point scale in which 1 = strongly disagree, and 5 = strongly agree. Exact items and response options are detailed in Table 1. Finally, demographic information was self-reported.

**Table 1:** Survey items.

	Survey items with distribution of responses					
VR adjustment	About how long did it take for you to adjust to the VR environment?	1–2 min (60.0%)	3–4 min (17.5%)	5 or more minutes (15.0%)	I never adjusted to the VR environment (7.5%)	
Cybersickness	Did you experience any discomfort while in the environment?	1-none at all (37.5%)	2 (32.5%)	3-some (27.5%)	4 (2.5%)	5-a great deal (0%)
	Did you experience any nausea while in the environment?	1-none at all (77.5%)	2 (15.0%)	3-some (2.5%)	4 (2.5%)	5-a great deal (2.5%)
	Did you experience any dizziness while in the environment?	1-none at all (55.0%)	2 (27.5%)	3-some (12.5%)	4 (5.0%)	5-a great deal (0%)
	Did you experience any eye strain either while in the environment or after removing the VR headset?	1-none at all (55.0%)	2 (27.5%)	3-some (12.5%)	4 (5.0%)	5-a great deal (0%)
Visual realism	The environment seemed realistic and believable	1-strongly disagree (0%)	2 (12.5%)	3 (42.5%)	4 (32.5%)	5-strongly agree (12.5%)
Equipment comfort	The equipment was comfortable and easy to wear	1-strongly disagree (2.5%)	2 (2.5%)	3 (2.5%)	4 (42.5%)	5-strongly agree (50.0%)
Controller usability	The handheld control was easy to understand and use	1-strongly disagree (5.0%)	2 (7.5%)	3 (10.0%)	4 (25.0%)	5-strongly agree (52.5%)
Immersion	I felt immersed in the VR environment	1-strongly disagree (2.5%)	2 (2.5%)	3 (20.0%)	4 (42.5%)	5-strongly agree (32.5%)
Perceived safety	The environment felt safe	1-strongly disagree (0%)	2 (2.5%)	3 (5.0%)	4 (25.0%)	5-strongly agree (67.5%)
	I felt relaxed in the environment	1-strongly disagree (2.5%)	2 (2.5%)	3 (2.5%)	4 (40%)	5-strongly agree (52.5%)
Environment attractiveness	The environment was attractive	1-strongly disagree (0%)	2 (5.0%)	3 (30.0%)	4 (30.0%)	5-strongly agree (35.0%)
Perceived task difficulty	The tasks were easy to complete	1-strongly disagree (0%)	2 (5.0%)	3 (15.0%)	4 (55.0%)	5-strongly agree (25.0%)

### Procedure

Upon arrival at the study site, participants were given a brief explanation of what the study entailed, and they provided their informed consent. Once consent was obtained, a research assistant demonstrated how to wear and adjust each component of the VR backpack and HMD. Once the participants had adjusted the equipment so it was comfortable and a research assistant verbally confirmed that they could accurately see and hear the simulation, the participant was given one hand controller.

Following proper calibration of the equipment, participants were assured that the research assistant would be walking alongside them during the entire experience, and that the research assistant would redirect them if they approached a wall in the physical world by either tapping their shoulder or by verbal cues. Additionally, the research assistant explained that they would be carrying a hand controller, which can be seen in VR, so participants were always able to locate the research assistant during the experience. This procedure was created to assuage participants’ fears of colliding with the physical world.

Participants were trained in locomotion and manipulation of the VR environment. The research assistant showed the participant the two buttons (trigger button and thumbpad) on the hand controller that the participant was to use and confirmed with them that these were the only buttons they should use. They taught the participant to use the trigger button to teleport in the simulation and had the participant practice this maneuver. The participant was instructed that teleportation was only to be used if they accidentally entered a zone in the virtual environment from which they could not walk out (i.e., walking through a building enclosure). Next, the research assistant taught participants how to use the thumb touchpad, which could be pressed to rotate the virtual environment around the participant. This skill was necessary when participants encountered a physical environment barrier but wished to walk further in one direction in the virtual environment. The participant practiced this manipulation several times.

At this point, participants were reminded that if they felt uncomfortable at any time, they could remove the HMD and end their participation. A research assistant instructed participants to explore the virtual environment with the goal of completing three tasks; (1) find a crosswalk, (2) find a restaurant where they would bring a friend from out of town, and (3) find a FedEx store. These tasks were chosen because they imitate behaviors performed in the real-world counterpart of the virtual environment (i.e., the historic district of a mid-size city), thereby boosting ecological validity. Specifically, crosswalks need to be located to navigate the region which contains a main thoroughfare. Finding a restaurant to bring a visiting friend requires choice on the part of the participant. That is, if they wanted to complete the tasks quickly, they could choose the first restaurant they saw; however, they could also choose to continue searching and find a restaurant with which they were more satisfied. In this way, this task allows us to probe walking decisions. Lastly, needing to find a specific location is a common task in the real world. We chose the FedEx because it is obscurely placed away from the main thoroughfare, making it difficult to locate.

While in the simulation, a research assistant followed the participants within arms-length in order to stop and redirect them. The dimensions of the study room were 194′ 4″ × 114′ 7″. When a participant reached a physical boundary line on the floor, the research assistant redirected them by tapping their shoulder or providing verbal cues. This boundary line varied from 3′ 5″ to 14′ 1″ in its distance to the wall, meaning participants should not have walked closer than three feet to any physical wall.

When participants completed all three tasks, the simulation portion of the study session ended. If after 20 min participants had not completed all tasks, they were asked to report how they were feeling and if they wanted to continue to explore the simulation. If a participant wanted to end their study session at this time, the research assistant helped the participant remove the equipment and directed them to a computer to complete the post-experience survey. If the participant wanted to continue to explore the virtual environment, they were allowed to do so for 10 additional minutes at which point the research assistant ended their session. Once their time in the simulation had ended, all participants were directed to a laptop computer on which they completed the post-experience survey. Following completion of the survey participants were thanked for their time and awarded course credit.

### Statistical analyses

Descriptive statistical analyses (means and standard deviations) were conducted in 2021 using RStudio Statistical Software version 1.2.1335 (RStudio Team, Boston, MA, USA) in order to determine the feasibility and acceptability of using virtual reality to experimentally evaluate environmental modifications. We tested two versions of the environment (one with added greenery and one without added greenery) but pooled all participants for the present descriptive analyses assessing acceptability and feasibility of the overall methodology, rather than the effects of a specific environmental intervention on walking, in accordance with the CONSORT 2010 feasibility guidelines which state that “a feasibility study is not a hypothesis testing study, and therefore, no inferential statistical test should be conducted”^98,99^.

As all the subjective rating scales were from 1 to 5, we combined the ratings of 1 with 2 and 4 with 5 when calculating the proportions of participants endorsing each direction for simpler interpretations. For example, in the question “Did you experience any nausea while in the environment,” we combined people rating 1 and 2 to calculate the proportion of participants who experienced little to no nausea in VR. For the question “The environment felt safe,” we combined people rating 4 and 5 to calculate the proportion of participants who felt safe when using the simulation.

### Ethics approval and consent to participate

This study was approved by Colorado State University’s Institutional Review Board (#3526). Written consent was obtained from all participants prior to enrollment. Informed consent was obtained to publish the information/image(s) found in Fig. 1 in an online open-access publication as it contains potentially identifying information.

## Results

Forty participants (60% female; mean age 18.7 years) completed the walking task and all survey measures. The average time spent in the simulation was 19.22 min. Participants responded favorably to both the equipment and the VR environment created for this study: 92.5% felt equipment was comfortable to use; 92.5% felt safe when using the simulation, and 92.5% felt relaxed. Only 2.5% of participants reported experiencing significant discomfort during the study; 92.5% reported little to no nausea; 82.5% reported little to no dizziness, and 82.5% reported little to no eye strain. Only 5% found the environment unattractive, and 75% felt immersed in the simulation. For the vast majority of participants (77.5%), less than 5 min were needed to adjust to the simulation. See Table 1 for complete response information. Participant demographic information can be found in Table 2.

**Table 2:** Participant demographic characteristics.

Participant demographics (n = 40)	
Gender (% female)	60%
Mean age (in years)	18.7 (*SD* = 1.38)
Handedness (% right)	93%
Height (% 5′0–5′8)	65%
Height (% 5′9–6′2)	35%

## Discussion

This study examined the feasibility and acceptability of a novel VR approach to experimentally test the impacts of environmental modifications on walking behavior. In this study, it was possible to recruit participants, to create and modify the virtual environment in VR, and to provide participants with sufficient space for them to walk safely through the VR model, using overground walking. In addition, the overwhelming majority of participants reported feeling comfortable, safe, relaxed, and immersed; together, we believe these results constitute preliminary evidence that this VR approach to testing the effects on walking of modifying environments is both feasible and acceptable.

### Strengths

Superior to common methods identifying relationships between the environment and PA, VR technology provides the possibility to assess causation due to its high degree of experimental control^94^. To assess walkability, participants need to walk freely through VR without feeling uncomfortable. Dizziness, headache, and motion-sickness have been reported by VR users in other contexts^100^. A systematic review of VR used in managing acute pain and anxiety for inpatients suggests that these and similar side effects of using VR occur in 0.5–8% of VR users^101^. Consistent with this estimate, only one of our participants (i.e., 3%) reported experiencing significant discomfort. Regarding specific side effects, 7.5% of our participants reported experiencing some nausea, 13% reported dizziness, and 18% reported eye strain. As previous research on side effects of VR has primarily focused on sitting or standing tasks, it is possible that walking freely while in VR may lead to a relatively higher incidence of some of the common VR side effects. One study assessing simulator sickness and postural stability through VR while using a treadmill suggests that sickness symptoms, including dizziness, headache, and eyestrain, were mild, and participants could still successfully complete the session^102^. Similarly, while walking freely, participants in our study were able to complete assigned tasks despite the slightly higher self-reported rate of side effects. Together with the low rate of significant discomfort, our results suggest that walking freely in VR is a viable modality to assess environmental impacts on walkability.

Subjectively-experienced immersion is another critical element to VR user-experience. Virtual reality therapy (VRT) is used as a clinical therapeutic intervention because of its ability to provide a sense of realism, thus promoting interest and encouraging participation^103^. The sense of realism largely contributes to the ecological validity of VR studies. Through multisensory stimuli with experimental control, participants can respond realistically in the virtual environment as if they were in reality^104^. In our study, 75% of participants reported the feeling of immersion as measured by agreement with the survey item “I felt immersed in the VR environment.” This high level of reported immersion indicates this simulation is ecologically valid and can be used for future studies to assess how modifying this environment affects walking.

### Limitations

Although promising, this study is not without its limitations. First, we did not collect data regarding if participants stopped walking because they had finished all tasks or because they wanted to end their session for any other reason. Presumably, if a participant wanted to end their session because of some negative feeling, our post-experience survey should capture it. Still, future research should differentiate between those who complete the VR session and those who ended the session without completing all tasks. Second, the participants were all healthy college students with an average age of 18.7 years, and all were residing in one geographic area. We cannot generalize our results of subjective VR experience to a more diverse population. Third, we chose to use short measures to capture constructs such as immersion and cybersickness in the interest of respondent fatigue, yet more complex measures are available^105,106^. Also, since presence is a component of immersion^51^, we decided to measure only immersion and presume that improved immersion likely would have increased participants’ sense of presence. Our data could be improved by assessing the two related constructs separately. Furthermore, the physical space where this study was conducted was large, but not an unlimited space. Participants sometimes needed to turn their bodies and then rotate the simulation separately in the real world but not in the simulation.

### Ethical challenges of walkability research using VR

There are several ethical considerations that researchers face when conducting walkability research using VR methods. First, participants can experience fear when the virtual and physical environments do not align regarding obstacles, as demonstrated in Mousas and colleagues study using physiological (i.e., higher arousal) and behavioral measures (i.e., shorter steps)^92^. In the present study, we have taken clear precautions to reduce participant fear, including having participants walk in a large open space with no barriers in the center, and allowing the participant to locate the research assistant at all times through a visual cue in the virtual environment. Moreover, we included questions asking about the subjective feeling of safety and relaxation in our post-experience survey, and over 90% of participants expressed that they felt safe and relaxed in the environment.

Second, using VR could potentially expose participants to side effects (e.g., dizziness, nausea, eyestrain), and people who have not used VR before may be unaware they are prone to these side effects. Future researchers should be mindful when recruiting inexperienced VR users, or individuals who are prone to motion sickness or have visual impairments.

### Future directions

Future studies should continue to test how overground walking in VR and/or AR relates to walking in the real world with reference to various environmental factors. Toward informing more effective salutogenic design practices in urban environments, future research teams may consider using a setup similar to what we propose and collecting data regarding walking trajectories as these data may help elucidate the navigational decisions that participants make in VR as compared to real-world environments, such as choosing a more or less direct route to their destinations. Additionally, our methodology may be adapted to investigate relationships between walking perceptions, mechanics, physiology, and decisions rather than investigating one or two in isolation.

From the perspective of human behaviors, future research may look at the motivations for continuing walking in the virtual world. It is possible that some participants choose to spend more time in the simulation because this is new to them. Future research should limit the novelty effect of the virtual environment by recruiting experienced VR users, conducting multiple sessions, or adopting a longitudinal method. Additionally, our data show there is some variability in the time it takes participants to adjust to VR, but over three-quarters of participants felt adjusted after less than 5 min. We recommend future researchers include an adjustment period of at least 5 min after participants don the HMD before the study tasks begin to allow participants to get accustomed to the technology.

As VR technology develops, researchers should investigate methods of simulating nonvisual environmental factors that may affect walking decisions. Neo et al.^52^ list a number of these sensory experiences that affect humans but are difficult to reproduce in VR, including odor. At this time, it is not recommended that HMDs be used outside in sun as the direct light harm the lenses^107,108^. Thus, weather factors such as wind, temperature change, and heat cannot be naturally integrated into study designs. Researchers should explore ways to recreate these factors indoors.

Once this relationship is well understood, future VR and/or AR research testing different environmental modifications (i.e., changing elements such as sidewalk width, greenery, street lighting, bike lanes, etc.) can be understood in the context of their impacts on real-world walking behavior. Lastly, researchers should seek to test these modifications with diverse groups of participants to determine if there are demographic differences that moderate the impact of each environmental modification.

## Conclusions

The results of this study demonstrate the feasibility and acceptability of our approach using wearable VR technology to experimentally assess how walking behavior is affected by environmental changes. This study both supports and expands on the current literature by providing proof of concept for the utility of this VR approach to enable researchers to make causal claims about environmental design modifications and their impact on walking behavior. This novel approach for testing the behavioral impacts of environmental changes appears promising.

## Data Availability

The datasets generated and/or analyzed during the current study are available in the Open Science Framework repository, https://www.osf.io/z28jw/.
